# Mutually exclusive expression of *DLX2 *and *DLX5/6 *is associated with the metastatic potential of the human breast cancer cell line MDA-MB-231

**DOI:** 10.1186/1471-2407-10-649

**Published:** 2010-11-25

**Authors:** Monica Morini, Simonetta Astigiano, Yorick Gitton, Laura Emionite, Valentina Mirisola, Giovanni Levi, Ottavia Barbieri

**Affiliations:** 1 Department of Experimental Medicine, University of Genova, Largo R. Benzi 10, 16132 Genova, Italy; 2 Department of Neuroscience and Brain Technology, The Italian Institute of Technology, Via Morego 30, 16163 Genova Italy; 3 Embryogenesis and Tumorigenesis in Animal models, National Institute for Cancer Research, Largo R. Benzi 10, 16132 Genova, Italy; 4 UMR7221 CNRS/MNHN, Evolution of Endocrine Regulations, 7 rue Cuvier, 75231 Paris, Cedex 05, France; 5 Functional Genomics, National Institute for Cancer Research, Largo Rosanna Benzi 10, 16132 Genova, Italy

## Abstract

**Background:**

The *DLX *gene family encodes for homeobox transcription factors involved in the control of morphogenesis and tissue homeostasis. Their expression can be regulated by Endothelin1 (ET1), a peptide associated with breast cancer invasive phenotype. Deregulation of *DLX *gene expression was found in human solid tumors and hematologic malignancies. In particular, *DLX4 *overexpression represents a possible prognostic marker in ovarian cancer. We have investigated the role of *DLX *genes in human breast cancer progression.

**Methods:**

MDA-MB-231 human breast carcinoma cells were grown in vitro or injected in nude mice, either subcutaneously, to mimic primary tumor growth, or intravenously, to mimic metastatic spreading. Expression of *DLX2*, *DLX5 *and *DLX6 *was assessed in cultured cells, either treated or not with ET1, tumors and metastases by RT-PCR. *In situ *hybridization was used to confirm *DLX *gene expression in primary tumors and in lung and bone metastases. The expression of *DLX2 *and *DLX5 *was evaluated in 408 primary human breast cancers examining the GSE1456 and GSE3494 microarray datasets. Kaplan-Meier estimates for disease-free survival were calculated for the patients grouped on the basis of *DLX2*/*DLX5 *expression.

**Results:**

Before injection, or after subcutaneous growth, MDA-MB-231 cells expressed *DLX2 *but neither *DLX5 *nor *DLX6*. Instead, in bone and lung metastases resulting from intravenous injection we detected expression of *DLX5/6 *but not of *DLX2*, suggesting that *DLX5/6 *are activated during metastasis formation, and that their expression is alternative to that of *DLX2*. The *in vitro *treatment of MDA-MB-231 cells with ET1, resulted in switch from *DLX2 *to *DLX5 *expression. By data mining in microarray datasets we found that expression of *DLX2 *occurred in 21.6% of patients, and was significantly correlated with prolonged disease-free survival and reduced incidence of relapse. Instead, *DLX5 *was expressed in a small subset of cases, 2.2% of total, displaying reduced disease-free survival and high incidence of relapse which was, however, non-significantly different from the other groups due to the small size of the *DLX+ *cohort. In all cases, we found mutually exclusive expression of *DLX2 *and *DLX5*.

**Conclusions:**

Our studies indicate that *DLX *genes are involved in human breast cancer progression, and that *DLX2 *and *DLX5 *genes might serve as prognostic markers.

## Background

The abnormal expression of homeobox genes in many solid tumors and hematological malignancies [1,2] has reinforced the notion that these key regulators of embryogenesis can also play a role in neoplastic processes. *DLX *genes, the vertebrate homologues of *Drosophila distal-less (dll*), constitute a family of homeobox transcription factors involved in control of cell differentiation and morphogenesis. The mouse and human *DLX *gene system is formed by three bi-gene clusters: *DLX*1 and *DLX*2; *DLX*5 and *DLX*6; *DLX*3 and *DLX*4. All *DLX *genes are expressed by embryonic stem cells and play a role in the control of craniofacial embryogenesis [3], of neurogenesis [4], and of formation of the distal regions of extending appendages [5]. Furthermore, *DLX5 *and *DLX6 *are expressed in all developing bones and control osteoblastogenesis and osteoblast/osteoclast coupling Although *DLX *genes are expressed in several adult tissues, including bone, brain and epithelia, little is known about their possible involvement in neoplastic process. We have previously demonstrated [6,7] that *Dlx *genes participate to the regulatory cascade initiated by acute lymphoblastic leukemia *(ALL)-1 *gene, a master regulator gene whose disruption is implicated in human acute leukemias [8]. *DLX *genes respond differently to the t(4;11)(q21;q23) chromosomal abnormality: while the expression of *DLX*2, *DLX*3, and *DLX*4 is virtually abrogated, that of *DLX5 *and *DLX6 *is increased [8]. These data indicate that different members of the *DLX *gene family could play diverse roles in predisposing cells to leukemic transformation. Furthermore, evidences have been found on *DLX5 *involvement in T-cell lymphomas both in mouse models and in humans [9].

In non-hematological malignancies most of the available information concerns *DLX4*. *DLX4 *overexpression by ovarian cancer is strongly associated with high tumor grade and advanced disease stage [10]. This gene, when overexpressed in the breast cancer cell line MCF7, inhibits apoptosis [11]. However, recently published microarray studies demonstrated the upregulation of *DLX5 *in several human solid tumors, suggesting that overexpression of *DLX5 *could contribute to tumor progression and represent a novel prognostic marker [12,13]. The analysis of factors involved in *Dlx *gene regulation has evidenced that *Endothelin1 *(*ET1*) is directly involved in the activation of *Dlx5 *and *Dlx6 *and inhibits the expression of *Dlx2 *[14-16]. It is of note that ET1 expression has been directly associated to the invasive phenotype of breast tumor cells [17].

## Methods

### 2.1. Cell culture and ET1 treatment

MDA-MB-231 cells were obtained from Dr. Paola Manduca (Dipartimento di Oncologia, Biologia e Genetica, Università di Genova, Italy). Cells were grown in DMEM supplemented with 10% Fetal Calf Serum (FCS), 1% glutamine and 1% penicillin/streptomycin (all from Biochrom Seromed, Berlin, Germany) at 37°C in 5% CO_2 _atmosphere.

For ET1 treatment, adherent sub-confluent MDA-MB-231 cells were starved for 24 h in serum-free medium, trypsinized and centrifuged. Pellets were resuspended in serum-free medium either containing or not 100 nM ET1 (Sigma Aldrich, St. Louis, MO); cells were then plated into a 12-well tissue culture plate and incubated for 8 h. Total RNA was then isolated from each well using the RNeasy kit (QIAGEN, Hilden, Germany), according to manufacturer's instructions. All experiments were run in triplicate.

### 2.2. Animals

We used nude female mice of 5-6 weeks of age (Charles River, Calco, Italy) housed in laminar flow isolated hoods at the Animal Facility of the National Institute for Cancer Research in Genova. Housing and treatments of animals were in accordance with the Italian and European Community guidelines (D.L. 2711/92 No.116; 86/609/EEC Directive), and approved by the internal Ethic Committee.

### 2.3. Injection of MDA-MB-231 in mice

Sub-confluent MDA-MB-231 cells were trypsinized, resuspended in PBS and counted. Primary tumors were obtained by injecting 3 nude mice subcutaneously with 4 × 10^6 ^MDA-MB-231 cells. Nodules developed within 2-3 weeks and were dissected when they reached an approximate volume of 250 mm^3^, generally within 1 month from injection. Metastases were obtained by injection of 2 × 10^5 ^cells in 100 μl of PBS in the left cardiac ventricle of 10 nude mice. Mice were sacrificed 2 months after cell inoculation and subjected to necropsy. Before sacrifice bone metastases were identified by radiography. Nodules and metastases were immediately processed for total RNA extraction or paraffin embedding.

### 2.4. Human samples

We examined normal breast tissues from 2 women undergoing reductive mammoplastic and primary breast cancer tissues from 2 patients undergoing curative surgery, one for invasive ductal carcinoma and one for invasive mixed carcinoma. Samples were kindly provided by Dr. L. Mastracci (Dipartimento di Discipline Chirurgiche, Anestesiologiche, Morfologiche e Metodologie Integrate, Università di Genova, Italy), through the GTB (Genoa Tissue Bank). Written informed consent was obtained from all patients and approved by the S. Martino Hospital's Ethical Committee, Genova, Italy. This investigation conformed the principles outlined in the Declaration of Helsinki. *DLX2 *and *DLX5 *expression was investigated by RT-PCR.

### 2.5. Immunohistochemistry

All bones (hindlimbs, ribs and tail) were fixed overnight in 4% paraformaldehyde (PAF) at 4°C, washed in PBS, decalcified for 18-24 hrs in Osteodec (Bio-Optica, Milano, Italy) and embedded in paraffin. All other organs were fixed in 4% PAF at 4°C, washed in PBS and embedded in paraffin. For immunohistochemistry staining, de-waxed sections were treated in microwave oven for 15 min in 10 mM citrate buffer pH 6.0 and incubated with 3% H_2_O_2 _for 10 min. After 1 hr in blocking solution (10% normal goat serum in PBS), sections were incubated for 40 min at room temperature with either a rabbit monoclonal anti-Ki67 (1:250) or a mouse monoclonal anti-Keratin 18 (K18) Ab-8 clone L2A1 (1:100) (NeoMarkers, Fremont, CA). After washing with PBS, sections were incubated with the appropriate secondary biotinylated antibody (BIO-SPA, Milano, Italy) for 30 min at room temperature followed by 10 min incubation with horseradish peroxidase (HRP)-conjugated streptavidin (BIO-SPA). HRP enzymatic activity was then revealed with diaminobenzidine (DAB).

### 2.6. In situ hybridization

8 μm sections were de-waxed, rehydrated, incubated in 10 μg/ml Proteinase K for 20 min at 37°C, and fixed with 4% PAF for 15 min at room temperature (RT). After washing in PBS, slides were incubated for 10 min in 0.2 M triethanolamine and 0.25% acetic anhydride, washed with PBS and incubated 15 min in PBS containing 1% Triton X-100. After washing, sections were prehybridized in 50% formamide, 5× SSC, 5X Denhardt's solution, 0.25 mg/ml yeast tRNA, 0.5 mg/ml salmon sperm DNA (all from Sigma). Overnight hybridization was performed at 60°C in prehybridization buffer containing 0.5 μg/ml probe. *Dlx5 *and *Dlx6 *probes were kindly provided by Dr. Giorgio Merlo (Università di Torino, Italy). Slides were washed in 5X SSC 5 min at 60°C, 1 hr at 60°C with 0.2 XSSC, 5 min in 0.2X SSC at RT and then incubated for 1 hr with blocking solution containing 10% goat serum in 0.1 M Tris pH 7.5, 0.15 M NaCl at RT in a humidified chamber. Alkaline Phosphatase-conjugated anti-digoxigenin antibody (Roche Biochemicals, Basel, Switzerland) in blocking solution was then added to slides and incubated overnight at 4°C in a humidified chamber. Finally slides were washed in a solution containing 0.1 M Tris pH 7.5, 0.15 M NaCl, equilibrated 5 min in a solution containing 0.1% Tween 20, 0.1 M Tris pH 9.5, 0.1 M NaCl, 0.05 M MgCl_2, _1 mM levamisole and stained with BMPurple (Roche). Negative controls were run in parallel using sense probes.

### 2.7. RT-PCR

Total RNA was isolated from 70-80% confluent MDA-MB-231 cells and from human and murine tissue samples using the RNeasy mini kit (QIAGEN). Reverse transcription of 1 μg of DNase I-treated total RNA was performed using a Mo-MLV RNase-H(-) RT (Promega, Madison, WI) in a reaction volume of 20 μl. Resulting cDNA was then diluted by addition of 30 μl of water to reach a final volume of 50 μl and a concentration of cDNA 10×. Two μl of cDNA were used for amplification using Taq*DNA *polymerase (TibMolBiol, Genova, Italy).

The following primers were used:

human *DLX5 sense*   5'-ACCATCCGTCTCAGGAATCG

human *DLX5 antisense*   5'-ACCTTCTCTGTAATGCGGCC

human *DLX6 sense*   5'-GAACTGGCAGCTTCCTTA

human *DLX6 antisense*   5'-ACCAGTGAGAATAGCCAG

human *DLX2 sense*   5'-CTCTGCCTGCCTCATAAGG

human *DLX2 antisense*   5'-ATCGTAAGAACAGCGCAACC

human *G3PDH sense*   5'-GAAGGTGAAGGTCGGAGT

human *G3PDH antisense*   5'-TGGTTTCCGGACTTTCTTTG

murine *β-actin sense*   5'-TCGTGGGCCGCTCTAGGCAC

murine *β-actin antisense*   5'-TGGCCTTAGGGTTCAGGGGG.

The primers used for RT-PCR where specific for the human *DLX *genes and did not recognize mouse *Dlx*s. Annealing temperature was set to 60°C in all case, using 25 cycles for G3PDH and β-actin, and 35 cycles for the other genes.

### 2.8. Microarray data analysis

Expression analyses of human breast cancers were performed on the dataset GSE1456 [17] and GSE3494 [18] obtained from Gene Expression Omnibus http://www.ncbi.nlm.nih.gov/geo. The data sets consists of 159 and 249 breast cancers from unselected consecutive patients who received surgery at Karolinska Hospital from 1994-1996 or at the Uppsala County Hospital from 1987-1989, respectively. Expression sets were calculated from raw microarray of the two combined datasets using the GCRMA algorithms implemented in Bioconductor [19]. The threshold level of positive gene expression was set to five on the log2-scale [20]. GeneSpring 7.1 (Agilent, Santa Clara, CA) software was used for visualization of expression data. Samples were clustered according to the expression of *DLX2 *and *DLX5 *to enhance visual interpretation by hierarchical clustering with average linkage. Kaplan-Meier curves for disease-free survival were calculated using the package "survival" of Bioconductor [19].

## Results

### 3.1. DLX5, DLX6 and DLX2 expression in MDA-MB-231 cells, primary tumors and metastasis

**Figure 1:**
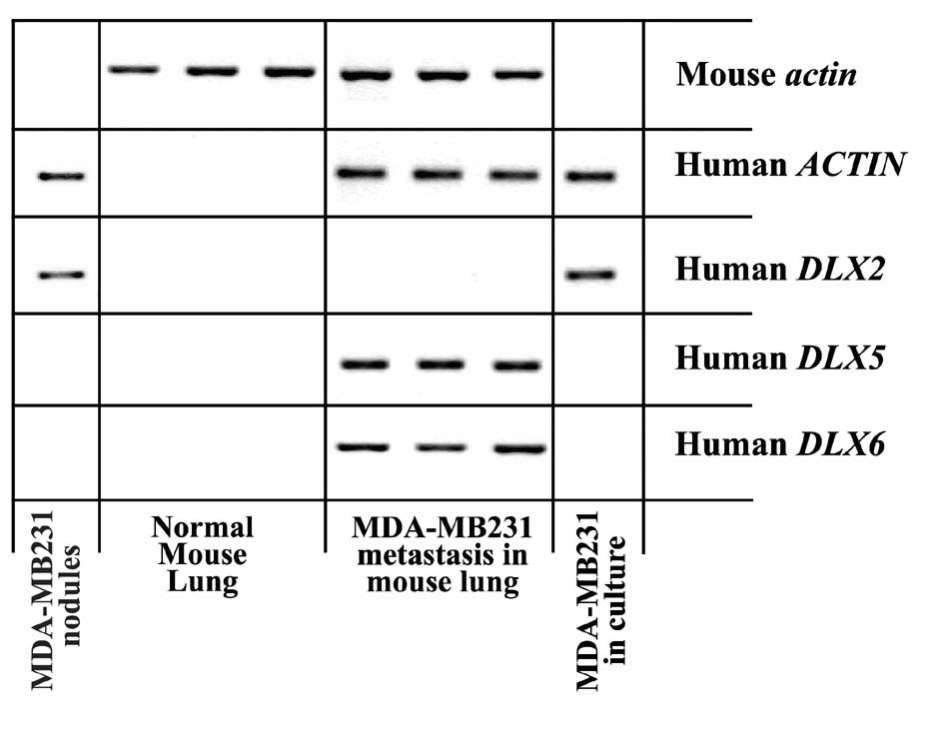
**Expression of *DLX2*, *DLX5 *and *DLX6 *in MDA-MB231 cells *in vitro *and *in vivo***. The pattern of expression of *DLX2, 5 *and *6 *in cultured MDA-MB-231 cells paralleled that observed in nodules resulting from subcutaneous injection into nude mice: a strong *DLX2 *expression was found, while neither *DLX5 *nor *DLX6 *were present. Lung metastases generated by injection of MDA-MB-231 into the left cardiac ventricle of nude mice displayed the opposite pattern of expression, with undetectable levels of *DLX2 *and high levels of *DLX5 *and *6*.

**Figure 2:**
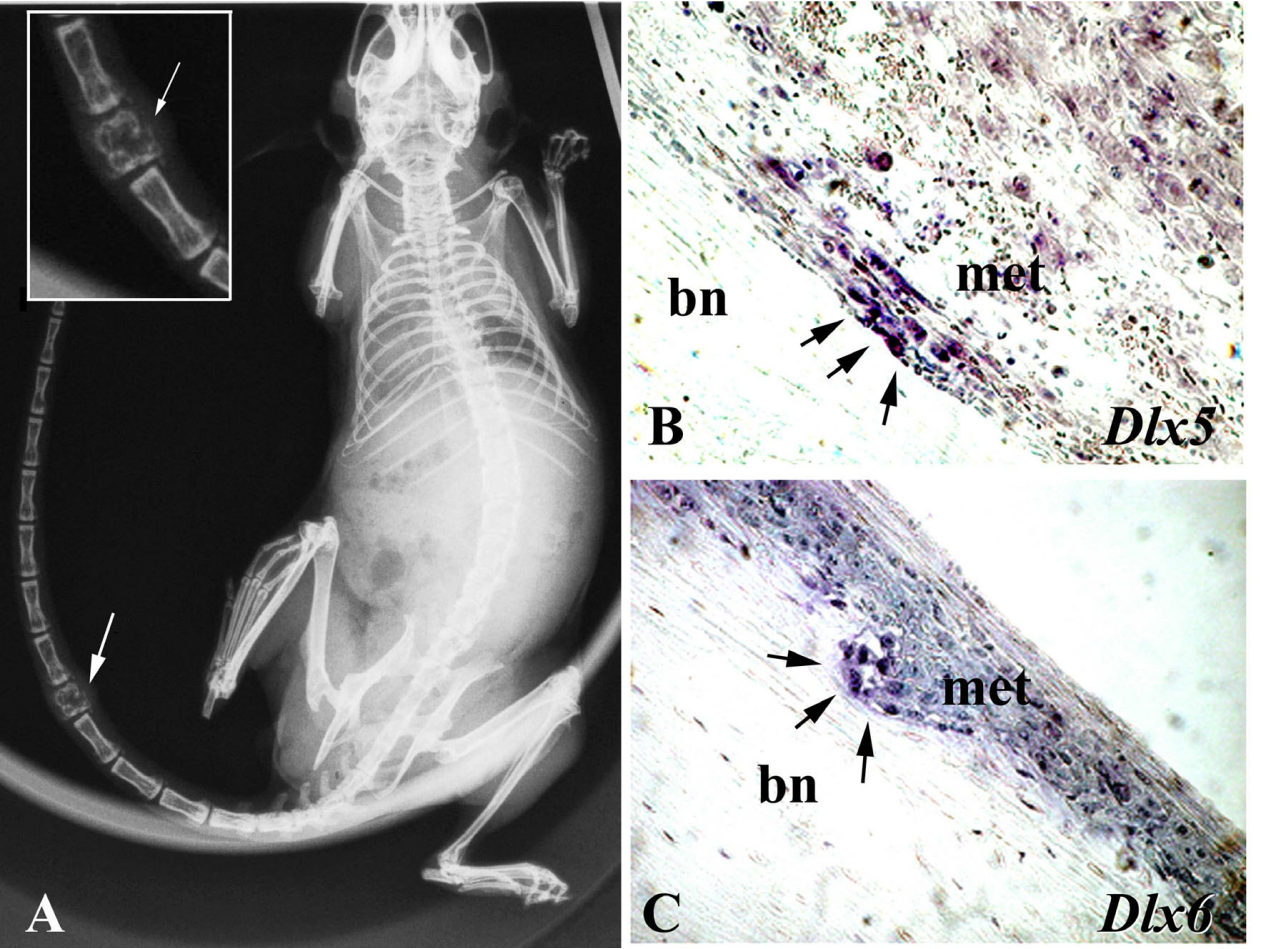
***In situ *hybridization for *DLX5 *and *DLX6 *in MDA-MB-231 bone metastases**. (A) A bone metastasis, visualized by radiography. (B and C) In-situ hybridization on sections of long bones containing MDA-MB-231 metastases showing high expression of *DLX5 *and *DLX6*. The signal was more intense at the invasion front (arrows). bn, cortical bone; met, metastasis.

**Figure 3:**
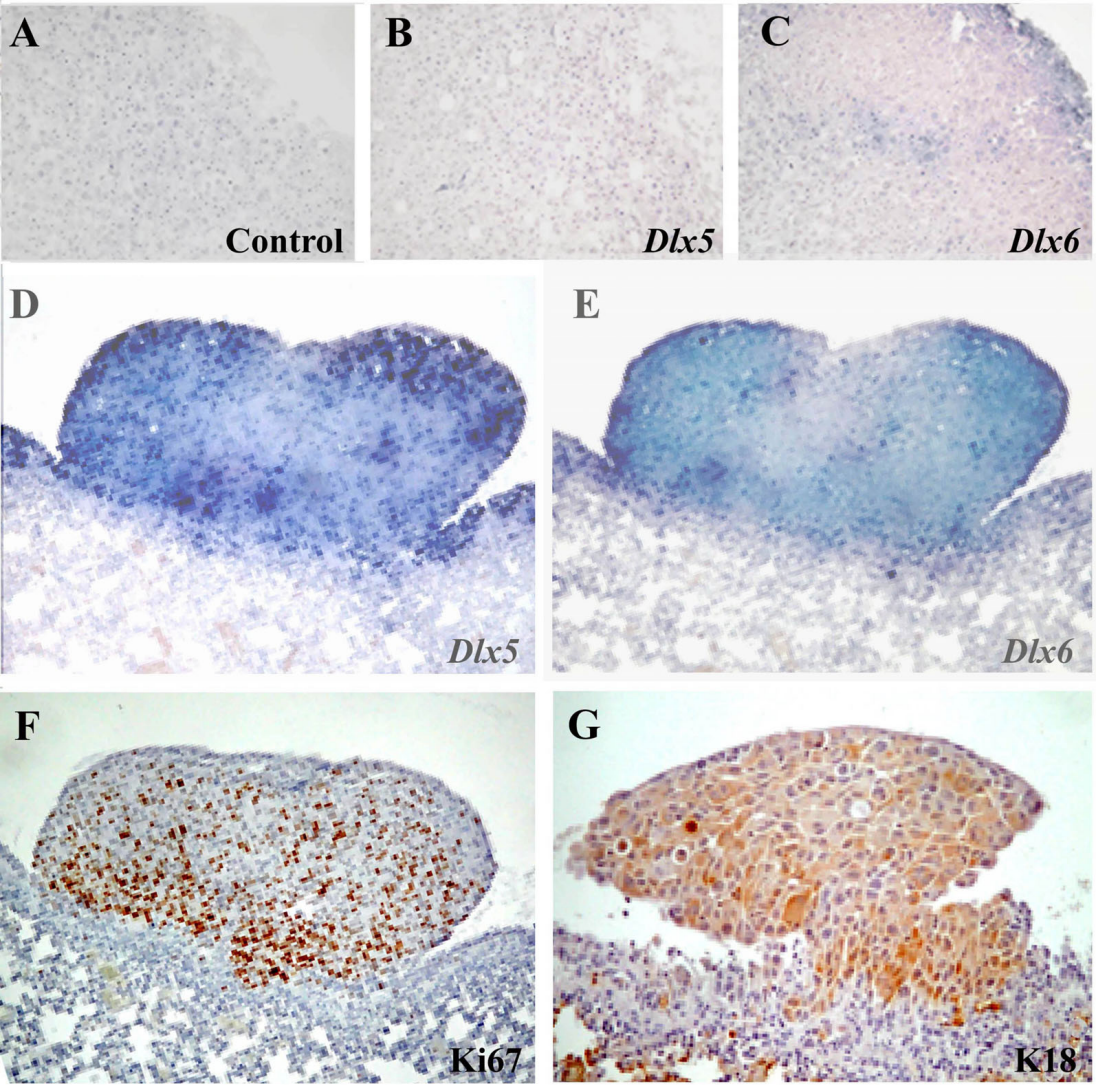
***In situ *hybridization for *DLX5 *and *DLX6 *in MDA-MB-231 lung metastases and subcutaneous nodules**. (A and C) Expression of *DLX5 *and *DLX6 *analyzed by *in situ *hybridization on sections of nodules derived by subcutaneous injection of MDA-MB231 cells in nude mice or (D and E) in sections of lung metastases derived from intracardiac injection of the same cells. (F) The proliferative state of the metastases was evidenced by Ki67 immunostaining. (G) K18 immunostaining of metastases was used to specifically identify human cells.

To analyze the role of DLX2, DLX5, and DLX6 in breast primary tumor and metastasis formation, we used an experimental model of breast cancer metastasis. The MDA-MB-231 human mammary carcinoma cell line gives rise to metastases in several organs, including bones, when inoculated into the left cardiac ventricle of immunocompromised mice [21]. Instead, when MDA-MB-231 cells are injected subcutaneously, a nodule grows at the site of injection, without significant metastatic spreading [22]. We first analyzed the expression of *DLX *genes by RT-PCR in MDA-MB-231 cells in sub-confluent culture, the same cell density used for *in vivo *experiments. We found that cells express *DLX2*, but neither *DLX5 *nor *DLX6 *(Figure 1). Then, to assess *DLX *gene expression in primary tumors, we injected MDA-MB-231 cells subcutaneously in nude mice. By RT-PCR analysis we found that subcutaneous nodules were positive for *DLX2*, but neither for *DLX5 *nor for *DLX6*, as the *in vitro *cultured cell line (Figure 1). Instead, a different expression pattern was found when the expression of *DLX2, DLX5*, and *DLX6 *was checked in lung and bone metastases growing from MDA-MB-231 cells injected into the left cardiac ventricle. Expression of *DLX5 *and *DLX6*, but not of *DLX2*, was found by RT-PCR in lung metastases, a result opposite to that obtained in cultured cells and subcutaneous nodules, suggesting that activation of *DLX5/6 *and suppression of *DLX2 *had taken place during metastasis formation (Figure 1). To obtain a confirmation, we analyzed by *in situ *hybridization the expression of *DLX5 *and *DLX6 *on tissue sections of lung and bone metastases. Metastases showed high levels of *DLX5 *and *DLX6 *expression (Figures 2B, C and 3D, E). It is interesting to note that *in situ *hybridization on bone metastases showed an increased *DLX5/6 *expression signal intensity in tumor cells in close contact with the bone tissue, suggesting a correlation between expression of these genes and the invasion front. *DLX5 *and *DLX6 *signals co-localized with that of human K18, identified by immunohistochemistry with a human-specific antibody not cross-reacting with mouse, confirming that *DLX*-expressing cells were indeed of human origin (Figure 3G). To assess if *DLX5/6 *expression was related to cell proliferation, sections adjacent to those used for hybridization were stained with an anti-Ki67 antibody that specifically identifies actively proliferating cells. We could not find any clear correlation between the staining obtained with *in situ *hybridization and the location of Ki67-positive nuclei (Figure 3F). Confirming the results obtained by RT-PCR, no expression of *DLX5 *or *DLX6 *was found in subcutaneous nodules (Figure 3B, C).

### 3.2. ET1 treatment of MDA-MB-231 cells induces DLX2 to DLX5 switch

**Figure 4:**
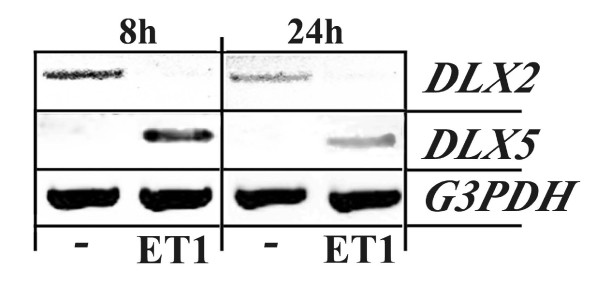
**ET1 treatment of MDA-MB-231 cells induces *DLX2 *to *DLX5 *switch**. Expression of *DLX2 *and *DLX5 *expression in of MDA-MB-231 cells treated with ET1. Subconfluent cultures were serum-starved and treated with vehicle alone (-) or ET1. RT-PCR analysis for *DLX2*, *DLX5 *and *G3PDH *was performed on total RNA isolated from individual wells.

During embryonic development ET1 plays a central role in the control of *Dlx *gene expression. In particular ET1 signaling is necessary and sufficient for the activation of *Dlx5 *in the mandibular arch [14,16]. ET1 expression has been directly associated to the invasive phenotype of breast tumor cells in mouse models, but cultured MDA-MB-231 cells do not express ET1 [23]. To test whether ET1 signaling could be at the origin of the switch in *DLX *gene expression observed during metastasis formation, starved MDA-MB-231 cells where treated with ET1. We observed a clear reduction of *DLX2 *expression, paralleled by induction of *DLX5 *(Figure 4), suggesting that ET1 signaling could be involved in the *DLX*2 to *DLX5 *switch observed in metastases.

### 3.3. DLX2 expression in human breast cancer is positively associated with disease-free survival

**Figure 5:**
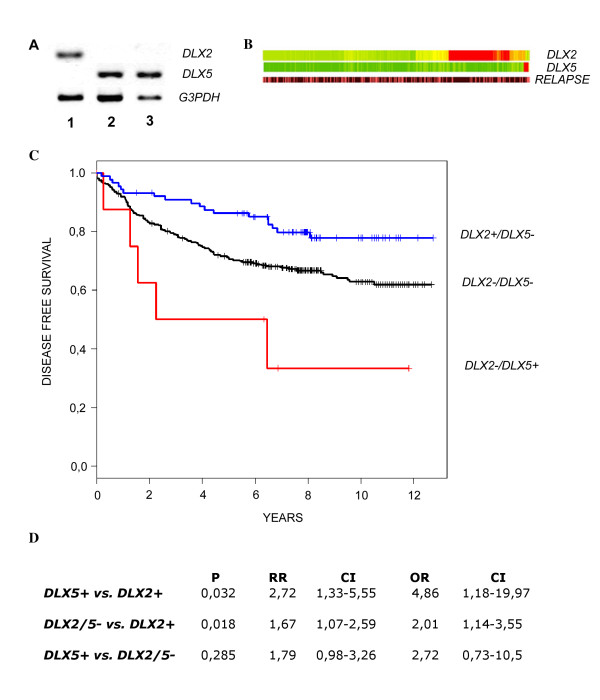
***DLX2 *and *DLX5 *expression and correlation with disease-free survival in human breast cancers**. (A) *DLX2 *and *DLX5 *expression were analyzed by RT-PCR in normal breast tissue (lane 1), ductal breast carcinoma (lane 2), and mixed breast carcinoma (lane 3). (B) Relative expression of *DLX2 *and *DLX5 *in 408 human breast cancer samples as analyzed by microarrays (Gene Expression Omnibus datasets GSE1459 and GSE3494). Expression values are color-coded, red: expression above the mean value, green: expression below the mean value. Intensity of colour correlates to the strength of up- or downregulation. Samples are ordered by hierarchical clustering based on the expression values of these two genes. Incidence of relapse is indicated in the lower bar (brown: no relapse, pink: relapse; 12 year follow-up). (C) Kaplan-Meier estimates of disease free survival of *DLX2+/DLX5- *patients (n = 88), *DLX2-/DLX5- *patients (n = 311), and *DLX2-/DLX5+ *patients (n = 9). (D) Statistical analysis of relapse incidence among the three groups. The incidence of relapse was 18/88 for *DLX2*+*/DLX5- *patients, 106/311 for *DLX2*-*/DLX5- *patients, and 5/9 for *DLX2*-*/DLX5+ *patients. P = p-value, RR = relative risk, OR = odds ratio, CI = confidence interval.

In order to assess the role of *DLX2 *and *DLX5 *gene expression also in humans, we performed a preliminary analysis of the *DLX *status on 2 samples of normal breast tissue and on 2 samples of primary breast tumor. Normal breast tissues expressed *DLX2 *but not *DLX5*. Instead, the two cases of mammary carcinoma were positive for *DLX5*, but negative for *DLX2 *(Figure 5A). These preliminary findings indicate that in humans, as in mice, expression of *DLX5 *could be related to metastatic capacity. To confirm this hypothesis, we analyzed the expression of *DLX *genes in a dataset of 408 cases of human breast cancers obtained from Gene Expression Omnibus, and correlated results with disease-free survival and relapse. In mammary tumors, the expression levels of *DLX1, DLX3, DLX4 *and *DLX6 *did not significantly differ from those observed in normal tissue (data not shown). Instead, *DLX2 *was expressed in 21.6% (88/408) of the tumors, while *DLX5 *expression was present in only 2.2% (9/408) of the tumors (Figure 5B). In all cases expression of the two genes was mutually exclusive, in no instance co-expression of *DLX2 *and *DLX5 *was observed. Patients were then divided into three groups, i.e. *DLX2+/DLX5-*, *DXL2-/DLX5+ *and *DLX2-/DLX5-*, and disease-free survival and relapse were assessed. The *DLX2+/DLX5- *patients had the longest average disease-free survival, whilst the *DLX2-/DLX5+ *patients had the shortest one. Kaplan-Meier analysis of disease-free survival showed a significant difference between the *DLX2*+*/DLX5- *and *DLX2*-*/DLX5- *groups (p = 0.018), however, the very small size of the *DLX2-/DLX5+ *sample resulted in non-significant difference when comparing this group of patients with either the *DLX2+/DLX5- *or the *DLX5/2- *groups (Figure 5C and 5D). Taking into account the age of patients as a co-variant did not modify these results.

## Discussion

Several homeobox genes are deregulated in a variety human tumors, and their deregulation appears to enhance cell survival and proliferation and to inhibit differentiation [2]. Among the *DLX *family of homeobox-containing genes, *BP1*, an isoform of *DLX4*, was found to correlate with poor prognosis in human breast cancer [24]. In the present study we specifically investigated the involvement of *DLX2 *and *DLX5/6 *in breast tumor progression.

In our mouse model, we found that *DLX5 *and *DLX6*, not expressed by MDA-MB-231 cells either cultured or grown subcutaneously, were specifically induced in metastases growing in bone or lung. On the contrary, *DLX2 *was expressed in cultured cells and in subcutaneous nodules, but not in bone or lung metastases. These results indicate that transition to metastatic phenotype is characterized by inhibition of *DLX2 *and induction of *DLX5/6 *expression. The expression of *DLX5/6 *in metastases of different tissues suggests that this phenomenon is independent from the microenvironment present at the site of metastasis formation. The lack of relationship with Ki67 expression indicates that in this model the expression of *DLX5/6 *is independent also from cell proliferation, though previous data indicated that Dlx5 contributes to increased cell proliferation in mouse thymic lymphomas [9].

A preliminary analysis on human samples showed inhibition of *DLX2 *and induction of *DLX5 *expression in breast cancer, as compared with normal breast tissue. Furthermore, analysis of microarray data on human breast tumors showed that breast cancer patients with *DLX2 *expression had on average better prognosis and less incidence of relapse, while *DLX5 *expression and *DLX2 *downregulation, was related to a subset of more aggressive tumors. In the data set we examined *BP1 *had not been studied, so we cannot confirm the previous finding of a relationship between the expression of this gene and poor prognosis in human breast cancer [24]. As in mice, shift from *DLX2 *to *DLX5 *expression seems to be related to progression toward an increasingly malignant phenotype. However, *DLX5 *expression in human breast tumors is not associated with expression of *DLX6*. This is not surprising: also in human lymphomas upregulation of *DLX5 *occurs without accompanying up-regulation of *DLX6 *[9].

We suggest that the alternative expression of *DLX2 *and *DLX5 *might be considered as markers of better and worse prognosis. It has to be noted that also in primary lung tumors *DLX5 *expression is associated with poor prognosis [25]. Both in our model system and in human tumors, the expressions of *DLX5 *and *DLX2 *were mutually exclusive, in agreement with other experimental data demonstrating that these genes reciprocally downregulate their expression in different territories [26,27]. The opposite expression pattern of *DLX2 *and *DLX5 *in tumors are also consistent with previous observations, showing that *DLX2 *is expressed at higher levels in tumor cell lines which are more sensitive to apoptotic induction by fenretinide, whereas *DLX5 *and *DLX6 *appear to segregate in a distinct functional compartment [28]. The involvement of *ET1 *signaling could be at the origin of this switch in *DLX *gene expression. Indeed, ET-1 plays a role in breast cancer progression: it is associated with invading regions of tumors and is more common in tumors with high histological grade and lymph node invasion [29]. Furthermore, serum ET-1 is increased in patients with lymph node metastases, compared to those with no lymph node involvement. On these bases, ET-1 receptor antagonists have been used to prevent metastatic spreading in animal tumor models [30,31]. However, clinical trials so far have failed to demonstrate a therapeutical effect of this class of drugs in human solid tumors [31,32]. Not surprisingly, ET-1 was not independently predictive or relapse-free and overall survival in breast cancer [33] and, in the dataset we used ET1 is broadly present. Indeed, ET-1 induction could occur at the site of metastases and not of primary tumors.

## Conclusions

Herein we show that a *DLX2*-to-*DLX5/6 *switch in gene expression occurs during metastasis formation by MDA-MB-231 human breast carcinoma cells injected into nude mice. This switch also occurs *in vitro *by treating MDA-MB-231 cells with *ET1*. In breast carcinoma patients, *DLX2 *expression is associated with increased disease-free survival, while expression of *DLX5 *is present in a small number of particularly aggressive cases. Expressions of *DLX2 *and *DLX5 *are mutually exclusive. Our data suggest that *DLX2 *and *DLX5 *are involved in human breast cancer progression, and that they might serve as good or poor prognostic markers, respectively. In particular *DLX5 *overexpression, together with concomitant *DLX2 *downregulation, could identify a subset of more aggressive cancers that would need a different treatment and a more careful follow-up. We suggest considering *DLX2 *and *DLX5 *as potential prognostic markers, provided that our results are confirmed in a different and broader cohort of patients.

## Competing interests

The authors declare that they have no competing interests.

## Authors' contributions

MM and SA carried out the in vivo experiments, PCR and the ISH. LE carried out the immunohistochemistry. YG carried out the cell cultures. VM performed the microarray data analysis. MM, AS, GL and OB conceived the study, participated in its design and drafted the manuscript. All authors read and approved the final manuscript.

## Pre-publication history

The pre-publication history for this paper can be accessed here:

http://www.biomedcentral.com/1471-2407/10/649/prepub
